# Supplementary material for: Proximate analysis and vitamin B contents of fresh-made, canned chickpea and broad bean dips commercially produced in Jordan
Source: PLoS One. 2024 Dec 31;19(12):e0311149. doi: 10.1371/journal.pone.0311149 (PMC11687860; doi:10.1371/journal.pone.0311149)
Supplement: Attachment — Submitted filename: Response to Reviewers 1.docx [file pone.0311149.s002.docx]

**Response to Editor and Reviewer’s Comments**

**Manuscript Title:** Proximate Analysis and Vitamin B Contents of Fresh-made and Canned Chickpea and Broad Bean Dips Commercially Produced in Jordan

To Whom It May Concern:

We wish to thank the editor and reviewers for their favorable review and constructive comments.

| **Comments** | **Response** |
| --- | --- |
| **Journal requirements:**  **1.** When submitting your revision, we need you to address these additional requirements. Please ensure that your manuscript meets PLOS ONE's style requirements, including those for file naming. The PLOS ONE style templates can be found at https://journals.plos.org/plosone/s/file?id=wjVg/PLOSOne_formatting_sample_main_body.pdf and https://journals.plos.org/plosone/s/file?id=ba62/PLOSOne_formatting_sample_title_authors_affiliations.pdf. | We want to thank the editor for the valuable comments and feedback. The following revisions have been made in the revised manuscript.  **1.** Thank you. Based on your comment, we have gone through the manuscript and ensured that it meets PLOS ONE's style requirements, including those for file naming. |
| **2.** In your Methods section, please provide additional information regarding the permits you obtained for the work. Please ensure you have included the full name of the authority that approved the field site access and, if no permits were required, a brief statement explaining why. | **2.** Thank you. Based on your comment, we have added a statement regarding the permits we obtained for the work in the **Materials and Methods** **Section**. This statement was as follows:  “We communicated by email or phone with the most commercialized factories and restaurants specialized in chickpea and broad bean dips in Jordan (7 factories, 7 restaurants). Afterward, the research team visited several processing factories and restaurants producing chickpea and broad bean dips, as well as several hospitals and hotels. Only three processing factories and three restaurants agreed to participate in the study after explaining the study’s objectives, while the rest apologized.” Please see the track changes in **Materials and Methods Section** page 5, lines 94-102. |
| **3.** Thank you for stating the following financial disclosure: The Deanship of Research and Postgraduate Studies in Petra University. Please state what role the funders took in the study. If the funders had no role, please state: "The funders had no role in study design, data collection and analysis, decision to publish, or preparation of the manuscript." If this statement is not correct you must amend it as needed. Please include this amended Role of Funder statement in your cover letter; we will change the online submission form on your behalf. | **3.** Thank you. Based on your comment regarding the funder’s role is as follows:  "**The funders had no role in study design, data collection and analysis, decision to publish, or preparation of the manuscript."** |
| **4.** Thank you for stating the following in the Acknowledgments Section of your manuscript: We would like to express our deepest appreciation and gratitude to The Deanship of Research and Postgraduate Studies in Petra University for their continuous support. We also wish to thank all the restaurants and the factories for their kind help and assistance in this study. We note that you have provided funding information that is not currently declared in your Funding Statement. However, funding information should not appear in the Acknowledgments section or other areas of your manuscript. We will only publish funding information present in the Funding Statement section of the online submission form.  Please remove any funding-related text from the manuscript and let us know how you would like to update your Funding Statement. Currently, your Funding Statement reads as follows:  The Deanship of Research and Postgraduate Studies in Petra University. Please include your amended statements within your cover letter; we will change the online submission form on your behalf. | **4.** Thank you. Based on your comment, we have removed the sentence” *We would like to express our deepest appreciation and gratitude to The Deanship of Research and Postgraduate Studies in Petra University for their continuous support*” from the **Acknowledgments Section**. Please see the track changes in **Acknowledgments Section** page 22, lines 437-439. |
| **5.** We note that your Data Availability Statement is currently as follows: All relevant data are within the manuscript and its Supporting Information files. Please confirm at this time whether or not your submission contains all raw data required to replicate the results of your study. Authors must share the “minimal data set” for their submission. PLOS defines the minimal data set to consist of the data required to replicate all study findings reported in the article, as well as related metadata and methods (https://journals.plos.org/plosone/s/data-availability#loc-minimal-data-set-definition).  For example, authors should submit the following data:  - The values behind the means, standard deviations and other measures reported;  - The values used to build graphs;  - The points extracted from images for analysis.  Authors do not need to submit their entire data set if only a portion of the data was used in the reported study.  If your submission does not contain these data, please either upload them as Supporting Information files or deposit them to a stable, public repository and provide us with the relevant URLs, DOIs, or accession numbers. For a list of recommended repositories, please see https://journals.plos.org/plosone/s/recommended-repositories.  If there are ethical or legal restrictions on sharing a de-identified data set, please explain them in detail (e.g., data contain potentially sensitive information, data are owned by a third-party organization, etc.) and who has imposed them (e.g., an ethics committee). Please also provide contact information for a data access committee, ethics committee, or other institutional body to which data requests may be sent. If data are owned by a third party, please indicate how others may request data access. | **5.** Thank you. Based on your comment, we have added a supplementary file containing data used in this study. |
| **Additional Editor Comments:**  **1.** Please clarify Table 2 & 4, 1 & 3. What is the different information there? | **1.** Thank you. Tables 1 & 3 are the comparison between the three fresh samples alone and the three canned samples (chickpeas dips (Table 1) and broad bean dips (Table 3)). Whereas Tables 2 & 4 are comparison between fresh and canned (Table 1 for chickpea) and (Table 4 for broad bean dips). |
| **2.** All tables contain all margin. Please only put header and footer of the margin. | **2.** Thank you. Based on your comment, all tables were revised and only headers and footers were added. Please see the track changes for **Table 1** (Page 12), **Table 2** (Page 13), **Table 3** (Page 14), and **Table 4** (Pages 15). |
| **3.** Please mention your innovations in the manuscript. | **3.** Thank you for your suggestion. Based on your comment, the innovation(s) of this study were added to the **Discussion Section.** Please see the track changes in **Discussion Section** page 17, lines 324-328. |
| **4.** What is the practical application of your research? Please mention in the manuscript. | **4.** Thank you for your suggestion. Based on your comment, the practical applications of this study were added to the **Discussion Section.** Please see the track changes in **Discussion Section** page 17, lines 321-327. |
| **5.** Why sample size is so small? How could these samples make conclusion of the quality of products? | **5.** Thank you.  Many commercial factories, restaurants, hospitals, and hotels in Jordan which we approached for the study, declined participation, hence the sample size was small.  We also added an extra section titled **Limitation**, to point out the limitations of this study including the sample size. Please see the track changes in **Limitations Section** page 22, lines 420-424. |
| **6.** There are lots of brands in the market? How did you choose brands? | **6.** Thank you. The brands chosen for this study were based on: 1) Being the most commercialized brands produced and sold in Jordan, and 2) Being the products of the restaurants and factories which accepted to participate in this study. |
| **7.** How many batches were tested? | **7.** Thank you. Nearly 6 batches of chickpea dips and 6 batches of broad bean dips were selected randomly. Each batch contained 24 canned products. |
| **8.** Writing is so poor. Need to improve? | **8.** Thank you. Based on your comment, we have gone through the manuscript and made the improvements needed. |
| **9.** English is so poor. Need to improve? | **9.** Thank you. Based on your comment, we have gone through the manuscript and made the improvements needed. |
| **Reviewer's Responses to Questions:**  **1.** Is the manuscript technically sound, and do the data support the conclusions?  The manuscript must describe a technically sound piece of scientific research with data that supports the conclusions. Experiments must have been conducted rigorously, with appropriate controls, replication, and sample sizes. The conclusions must be drawn appropriately based on the data presented.  **Reviewer #1: Yes** | We want to thank the reviewers for the valuable comments and feedback. The following revisions have been made and highlighted (if applicable) in the revised manuscript.    **1.** Thank you. |
| **2.** Has the statistical analysis been performed appropriately and rigorously?  **Reviewer #1: Yes** | **2.** Thank you. |
| **3.** Have the authors made all data underlying the findings in their manuscript fully available?  The PLOS Data policy requires authors to make all data underlying the findings described in their manuscript fully available without restriction, with rare exception (please refer to the Data Availability Statement in the manuscript PDF file). The data should be provided as part of the manuscript or its supporting information, or deposited to a public repository. For example, in addition to summary statistics, the data points behind means, medians and variance measures should be available. If there are restrictions on publicly sharing data—e.g. participant privacy or use of data from a third party—those must be specified.  **Reviewer #1: Yes** | **3.** Thank you. |
| **4.** Is the manuscript presented in an intelligible fashion and written in standard English?  PLOS ONE does not copyedit accepted manuscripts, so the language in submitted articles must be clear, correct, and unambiguous. Any typographical or grammatical errors should be corrected at revision, so please note any specific errors here.  **Reviewer #1: Yes** | **4.** Thank you. |
| **5.** Review Comments to the Author.  Please use the space provided to explain your answers to the questions above. You may also include additional comments for the author, including concerns about dual publication, research ethics, or publication ethics. (Please upload your review as an attachment if it exceeds 20,000 characters).  **Reviewer #1:**  Dear author,  Thank you for the nice work. I have seen the detail of your work and my suggestion is please describe clearly the gaps why this finding is important and what gaps this finding will address. Discussion is shallow and please discuss in detail by using similar study conducted so far. | **5.** Thank you. Based on your comment, amendments addressing the **Discussion Section** have been made. Please see the track changes in the **Discussion Section** pages 17-21, lines 315-418. |

Thank you again. If there are any additional changes that you wish for us to make to the manuscript, do not hesitate to let us know.

Sincerely,

The Authors
